# Mondragon Unibertsitatea face-milling dataset for smart tool condition monitoring

**DOI:** 10.1038/s41597-025-05168-5

**Published:** 2025-05-23

**Authors:** Jose Joaquin Peralta Abadia, Mikel Cuesta Zabaljauregui, Felix Larrinaga Barrenechea

**Affiliations:** Mondragon Goi Eskola Politeknikoa, Faculty of Engineering, Arrasate, 20500 Spain

**Keywords:** Mechanical engineering, Industry, Technology, Scientific data, Computer science

## Abstract

This article presents a dataset of face-milling experiments for smart tool condition monitoring (TCM) performed under varying cutting conditions in the High-Perfomance Machining laboratory of Mondragon Unibertsitatea (MU). The experiments collected raw internal signals from the machine. Cutting forces, vibration signals, and acoustic emission signals were collected with external sensors. Tool wear was measured before each experiment and annotated accordingly, providing tool wear progression throughout the dataset. The dataset was technically validated using Python scripts to ensure the quality and reproducibility of the dataset. The resulting MU-TCM face-milling dataset offers a reproducible research design of experiments and associated data to carry out and advance smart TCM of milling processes. The dataset supports applications such as training machine learning and deep learning for TCM, enables sensor fusion research with diverse signal combinations, and facilitates the development of TCM solutions using only internal CNC signals for industrial environments. By supporting these applications, the dataset is expected to help reduce the gap between research and industry in smart TCM applications.

## Background & Summary

Machining processes, such as milling, form the backbone of modern manufacturing, particularly in the production of machine parts^1^. Maintaining the quality and performance of machining processes requires effective tool condition monitoring (TCM) systems, which track tool wear and performance in computer numerical control (CNC) machines. The introduction of such systems has led to an improvement in product quality, process reliability, and production efficiency.

In TCM, signals can be acquired from either external sensors, attached to the workpiece or spindle^2^, or internal sensors, placed on the table and spindles of CNC machines^3^. Although external sensors have proven to be effective in research environments, their complexity, cost, and difficulties in integration make them impractical for industrial applications, contributing to a significant gap between academic research and industry^4^. On the other hand, internal signals are easier to access, but are seldom employed in TCM because of their low resolution and restricted access, as commercial CNC systems often lack APIs or data extraction tools to retrieve the signals.

To overcome the limitations of both external and internal sensors, sensor fusion–the process of combining multiple sensor signals–has become a critical factor in improving the accuracy and reliability of TCM. Sensor fusion allows various aspects of machining processes to be monitored simultaneously, enhancing the quality of the data and improving overall process efficiency. This is particularly crucial in industrial settings, where internal signals such as motor torque, current, and power are more practical for implementation. By leveraging sensor fusion with internal signals, TCM systems can bridge the gap between academic research and industrial application, providing more reliable and efficient monitoring solutions for real-world manufacturing environments^4^.

A number of recent studies have proposed deep learning-based TCM systems (DL), which take advantage of the generalisation potential of DL knowledge^5,6^. Nevertheless, data collection for training DL models to date has been based on limited experiments or specific process conditions, since data collection and labelling are costly and time-consuming in machining processes. Moreover, training DL models for specific or limited process conditions widens the gap between research and industry. This is because DL models can only generalise knowledge from the data they are fed and are not easily transferable to other process conditions.

To reduce the burden of data collection during milling processes, a number of open-access datasets performing varied milling operations have been published for several monitoring scopes. Milling processes can differ significantly depending on the type of operation, such as face milling and side milling. These datasets were created by collecting data in both laboratory and industrial environments. The datasets collected in laboratory experiments focus on TCM and have limited ranges of cutting conditions and/or signal quantity^7–10^. The datasets collected in industrial environments, on the other hand, consider process health and workpiece quality monitoring^11,12^. These latter cover varying cutting conditions but collect only up to two signal types. Such a limited range of cutting conditions and signal types hinders the transfer of knowledge extracted from the datasets to industrial shop floors. In addition, some of these datasets were published more than one decade ago using what is now outdated equipment. Table 1 presents a comparison of the MU-TCM face-milling dataset with the milling datasets that focus on TCM.

**Table 1:** A comparison of the MU-TCM face-milling dataset with existing benchmark milling datasets.

Ref.	Operation	Experiments	Process conditions	Signals collected
Agogino and Goebel^7^	Face milling	16 (167 cuts)	- Material: Cast iron and stainless steel - Axial depth of cut (*a*_*p*_): 0.75 and 1.5 mm - Feed rate (*f*): 0.25 mm/rev and 0.5 mm/rev - Spindle speed (*n*): 826 RPM - Cutting speed (*v*_*C*_): 200m/min - Sampling rate: 250 Hz	- Acoustic emission, AE (table and spindle) - Vibration (table and spindle) - Current (AC and DC)
PHM 2010 data challenge^8^	Side milling	6 (945 cuts)	- Material: Stainless steel - *a*_*p*_: 0.2 mm - *f*: 1555 mm/min - *n*: 10,400 RPM - Sampling rate: 50,000 Hz	- AE (workpiece) - Vibration (workpiece) - Cutting forces (table)
Liu and Li^9^	Side milling	73 (199 cuts)	- Material: Titanium alloy (TC4) and superalloy (GH) - Tool material: Solid carbide and high speed steel - *f*: 0.021-0.08 mm/r - *n*: 820-2200 RPM - *a*_*p*_: 1-20 mm - Radial depth of cut (*a*_*e*_): 3 mm - Sampling rate: 600 Hz for force, 400 Hz for vibration, and 300 Hz for current and power	- Force (sensory tool holder in spindle) - Vibration (table) - Spindle current and power (PLC)
Denkena *et al*.^10^	Shoulder milling	9 (6418 cuts)	- Material: Cast iron 600-3/S - Three milling centers - *f*: 1,592 mm/min - *v*_*C*_: 200 m/min - *a*_*p*_: 2 mm - *a*_*e*_: 4 mm - Sampling rate: 25 kHz for external and 500 hz for CNC signals	CNC signals: - Position control deviation -Tool position (three axes) - Torque (spindle and three axes) - Force (three axes) External signals: - Force (three axes, table)
MU-TCM	Face milling	16 (67 cuts)	- Material: Cast iron and stainless steel - *a*_*p*_: 1.5 mm - *f*: 0.05, 0.1, and 0.2 mm/rev - *n*: 198.94, 397.89, 795.77 RPM - *v*_*C*_: 50, 100 and 200 m/min - Sampling rate: 1 Mhz for AE, 50 kHz for vibration and forces, and 250 Hz for CNC signals	- AE (table) - Vibration (three axes, table) - Force (three axes, table) - 16 internal CNC signals

This paper, therefore, presents the Mondragon Unibertsitatea-TCM (MU-TCM) milling dataset collected in a laboratory environment in the High-Perfomance Machining (HPM) laboratory of Mondragon Unibertsitatea (MU). Milling experiments were performed in a LAGUN L1000 CNC vertical machining centre. The dataset includes internal and external signals encompassing several cutting conditions and materials, and provides a balance between varying cutting conditions and signal variety. In addition, several high-frequency external signals and an extensive number of internal CNC signals were collected. The main contributions of the MU-TCM milling dataset to future TCM research are:

- Modern reproducible milling environment: The face milling experiments were performed in a modern CNC vertical machining centre using state-of-the-art milling cutters and workpiece materials. Thus, the knowledge that can be extracted from the dataset reflects modern industrial milling environments and can be reproduced.
- Sensor fusion applicability: The dataset includes an extensive number of external and internal CNC signals, which can assist in future identification of combinations of signals to exploit sensor fusion potential in TCM.
- Transfer and continual learning potential: The dataset is an ideal resource for developing and testing transfer learning and continual learning techniques for DL models. DL models can leverage the dataset to improve knowledge generalisation across a wide range of cutting conditions and operations, as well as continuously adapt to new data in milling environments.

The dataset approach is suitable for multiple applications, such as: training state-of-the-art machine learning and DL-based TCM systems for milling without the need for laboratory experiments, exploring the potential of sensor fusion with various combinations of signals, and training and testing DL-based TCM systems for industrial face milling applications with only internal CNC signals. Targeting these research efforts is expected to help reduce the gap between research and industry in TCM for machining. In addition, the dataset opens up new avenues for research in adaptive and resilient smart TCM systems.

## Methods

The MU-TCM dataset is a face-milling dataset for tool condition monitoring, specifically measuring tool wear. To create this dataset, a four-step methodology was defined: design of experiments (DOE), experimental setup, data collection, and data synchronisation.

### Design of experiments

The DOE for the MU-TCM dataset was based on industrial applicability and the recommended manufacturer settings of tools and workpiece materials. The DOE consisted of 8 combinations of cutting conditions, as presented in Table 2. Two materials were employed, cast iron (dry, without lubrication) and stainless steel (minimum quantity lubrication, MQL). The experiments were conducted at four cutting speeds *v*_*C*_ (100 and 200 m/mm for cast iron and 50 and 100 m/mm for stainless steel), and four feed rates *f* (0.1 and 0.2 mm/rev for cast iron and 0.05 and 0.1 mm/rev for stainless steel). The axial depth of cut *a*_*p*_ was 1.5 mm and the radial depth of cut *a*_*e*_ was 58.4 mm for all experiments.

**Table 2:** Design of experiments for the MU-TCM dataset.

Workpiece material	Lubication	*n* (RPM)	*a*_*p*_ (mm)	*a*_*e*_ (mm)	*v*_*C*_ (m/min)	*f* (mm/rev)
Stainless steel	MQL	198.94	1.5	58.4	50	0.05
Stainless steel	MQL	198.94	1.5	58.4	50	0.1
Stainless steel	MQL	397.89	1.5	58.4	100	0.05
Stainless steel	MQL	397.89	1.5	58.4	100	0.1
Cast iron	Dry	397.89	1.5	58.4	100	0.1
Cast iron	Dry	397.89	1.5	58.4	100	0.2
Cast iron	Dry	795.77	1.5	58.4	200	0.1
Cast iron	Dry	795.77	1.5	58.4	200	0.2

**Fig. 1:**
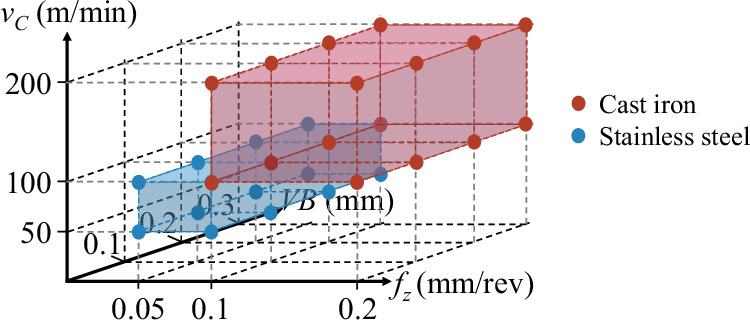
Three-dimensional view of the combination of cutting conditions and wear levels.

The procedure was designed to test four wear levels (0.0, 0.1, 0.2, and 0.3 mm) for each combination of cutting conditions, resulting in a total of 32 experiments. These wear levels were selected to simulate progressive tool degradation under realistic machining conditions. A three-dimensional representation of the experimental setup is illustrated in Fig. 1, with feed rate (*f*) on the *X* axis, cutting speed (*v*_*C*_) on the *Y* axis, and tool wear (*VB*) on the *Z* axis. Both materials were tested under an identical combination of cutting conditions, with a *v*_*C*_ of 100 m/min and an *f* of 0.1 mm/rev. This combination of cutting conditions was specifically designed to explore the potential for transfer learning and continual learning, by testing whether knowledge gained from one material can be applied to another.

### Experimental setup

**Fig. 2:**
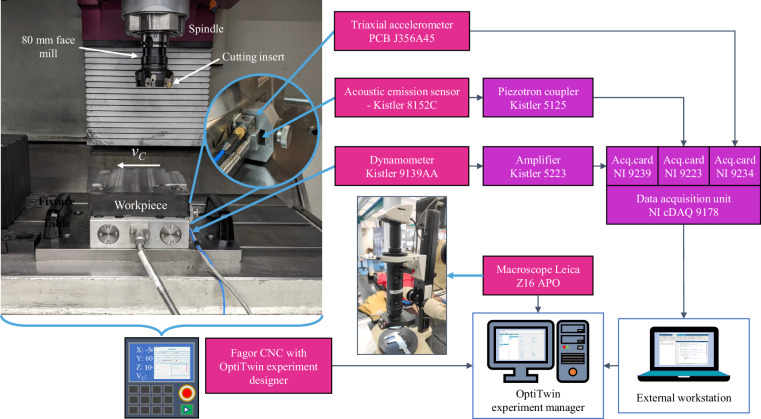
Experimental setup.

**Fig. 3:**
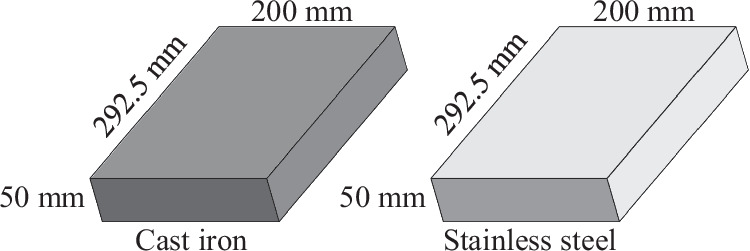
Dimensions of the materials used in the experiments.

Figure 2 illustrates the experimental setup. The experiments were performed in a LAGUN L1000 vertical machining centre with a Fagor CNC 8065 in the HPM laboratory of MU. An 80mm face mill with one Ayma SPKR M55 1203EDSR AFT720 cutting insert was used for material removal. Blocks of 200 × 292.5 × 50 mm (width x depth x height) were used for each material, as depicted in Fig. 3.

#### Signals

As shown in Fig. 2, three sensors were fixed to the table of the machining centre to acquire external signals:

- Cutting forces: A Kistler 9139 AA dynamometer was mounted to the table fixture and connected to a Kistler 5223 amplifier. Cutting forces were measured along the *X*, *Y*, and *Z* axes.
- Vibration: A PCB J356A45 triaxial accelerometer was coupled to the side of the dynamometer. Vibration signals were measured along the *X*, *Y*, and *Z* axes without pre-processing.
- Acoustic emission (AE): A Kistler 8152C AE sensor was coupled to the side of the dynamometer. The AE sensor was connected to a Kistler 5125 piezotron coupler, which amplified the signal and filtered it with high-pass (1 MHz) and low-pass (50 kHz) filters.

The dynamometer, AE sensor, and accelerometer outputs were then connected to an NI cDAQ 9178 data acquisition (DAQ) unit, via NI 9239, NI 9223, and NI 9234 analog-to-digital converter acquisition cards, respectively. The DAQ unit was connected to an external workstation via Ethernet, and the data was collected with a MatLab script. In addition, a root-mean-squared (RMS) function with a moving average was applied to the AE signal to reduce noise and emphasise significant signal trends, such as tool wear progression and cutting anomalies.

Internal CNC signals were acquired using the OptiTwin system^13^, in parallel with the external sensors. This system enabled the real-time collection of internal signals. The OptiTwin system consists of two key components: the experiment designer, which communicates with the Fagor CNC via a custom API, ensuring real-time data acquisition, and the experiment manager, which stores the internal CNC signals for further analysis. Table 3 summarises the details of the external and internal signals.

**Table 3:** Details of the internal (I) and external (E) signals. (*In contrast to the programmed value in the CNC.).

Signal ID	Sensor type	Type	Description	Units	Sampling freq. (Hz)	Range
SREAL	CNC	I	Actual spindle speed*	RPM	250	N/D
FREAL	CNC	I	Actual feed rate*	mm/rev	250	N/D
POS_S	CNC	I	Spindle angular position within a 360∘ rotation	^°^	250	N/D
POS_(X-Y-Z)	CNC	I	Spindle position relative to the machine table in three axes	*μ*m	250	N/D
TV50	CNC	I	Spindle motor power feedback	kW	250	±100 kW
TV51	CNC	I	Spindle motor active power	W	250	±2147 ⋅ 10^6^ W
CV3_(S-X-Y-Z)	CNC	I	RMS current feedback of the spindle motor and table motor in three axes	Arms	250	0–200 Arms
TV2_(S-X-Y-Z)	CNC	I	Motor torque feedback of the spindle motor and table motor in three axes	Nm	250	N/D
F_(x-y-z)	Kistler 9139AA	E	Cutting forces in three axes	N	50 ⋅ 10^3^	±20 ⋅ 10^3^N
A_(x-y-z)	PCB J356A45	E	Vibration (gravitational acceleration) in three axes	g	50 ⋅ 10^3^	±50g
AE_F	Kistler 8152C	E	Filtered acoustic emissions	V	10^6^	±10dB
AE_RMS	Kistler 8152C	E	RMS acoustic emissions	V	10^6^	±10dB

#### Tool wear measurement

**Fig. 4:**
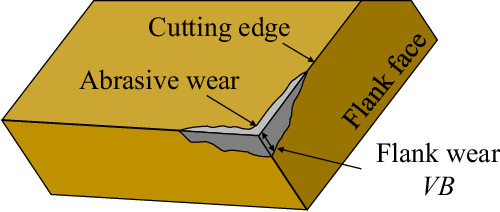
Flank wear *VB* on a cutting insert.

Tool wear takes various forms, such as cutting edge rounding, crater wear on the rake face, and flank wear due to friction. Flank wear *VB* was measured in this study, as it is a generally accepted parameter to evaluate tool wear^7^. *VB* is calculated as the distance from the cutting edge on the flank face of the tool to the end of the abrasive wear, as shown in Fig. 4. In these experiments, a Leica Z16 APO macroscope was used to measure the *VB* of the cutting inserts before each trial.

### Cutting process

**Fig. 5:**
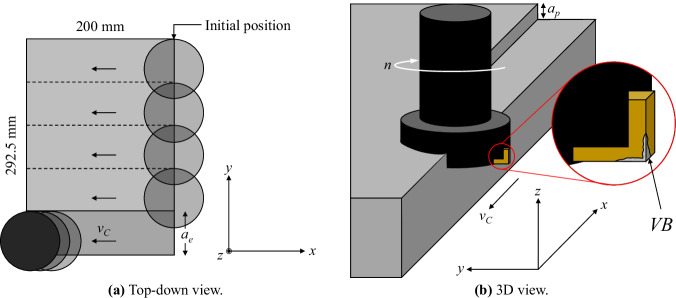
Experimental cutting process.

The cutting process consisted of horizontal face milling cuts along the *X* axis of the workpiece. Entrance cuts with a length of half the diameter of the face mill (40 mm) were made to prepare the workpiece and maintain consistent measurements throughout the experiments. This ensured that the tool would be in full contact with the material from the beginning to the end of each experiment. The cutting process is depicted in Fig. 5, with both a top-down view (Fig. 5a) and a 3D view (Fig. 5b). Axes are included to show the positioning of the material and the direction of the cutting speed (*v*_*C*_). A zoom-in of the *VB* has been added to Fig. 5b to demonstrate the location of flank wear during the cutting process.

**Fig. 6:**
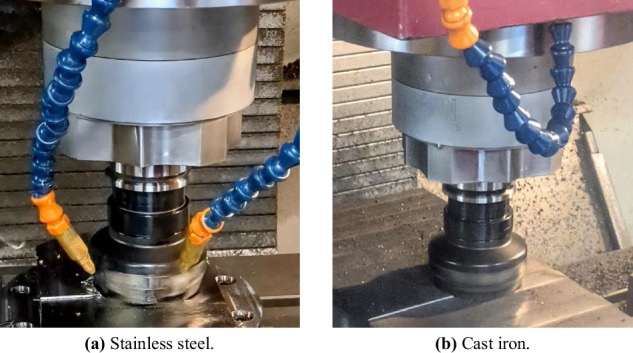
Actual cutting process examples for each material.

The defined radial depth of cut (*a*_*e*_) of 58.5 mm allowed for five complete passes through the workpiece with an axial depth of cut (*a*_*p*_) of 1.5 mm. This, together with the positioning of the face mill in the workpiece after the entrance cut, is illustrated in Fig. 5a. Figure 6 shows the cutting action with photographs of the actual cutting process for both stainless steel (Fig. 6a) and cast iron (Fig. 6b).

The face milling experiments were conducted in accordance with ISO-8688-1/1989 recommended tool life criteria: until tool wear reaches 0.3 mm or until fatal tool failure^14^. Table 4 details the number of experiments performed for each combination of cutting conditions and target *VB*, together with the total cutting time. Complete tool life experiments were not feasible due to practical constraints (time and cost). Therefore, to capture the behaviour of the tool across various wear stages efficiently, additional roughing cuts were performed to pre-wear the tool to approximate the next target *VB* value. This allowed for efficient data collection at various wear stages, although slight deviations existed between planned and actual values.

**Table 4:** Experiment count by cutting conditions and *VB*.

Material	*v*_*C*_ (m/min)	*f* (mm/rev)	Cut length (mm)	Cut time (s)	Experi-ment ID	Insert	Edge	Target *VB* (mm)	Real *VB* (mm)	Count	Total time (s)
Stainless steel 316L	50	0.05	3	18.1	1	9	1	0	0	2	36.19
					2	1	1	0.1	0.132	2	36.19
					3	3	1	0.2	0.202	3	54.29
					4	6	1	0.3	0.287	2	36.19
		0.1	3	9.05	5	9	1	0	0.03	2	18.1
					6	1	1	0.1	0.137	2	18.1
					7	3	1	0.2	0.206	2	18.1
					8	6	1	0.3	0.287	2	18.1
	100	0.05	3	9.05	9	9	2	0	0	2	18.1
					10	1	1	0.1	0.137	2	18.1
					11	3	1	0.2	0.206	2	18.1
					12	6	1	0.3	0.291	2	18.1
		0.1	4	6.03	13	9	2	0	0.039	2	12.06
					14	1	1	0.1	0.139	2	12.06
					15	3	1	0.2	0.213	2	12.06
					16	6	1	0.3	0.291	2	12.06
**Total**										33	355.88
Cast iron GG30	100	0.1	4	6.03	17	0	2	0	0	1	6.03
						0	3	0	0	1	6.03
					18	2	1	0.1	0.12	2	12.06
					19	4	1	0.2	0.201	2	12.06
					20	6	1	0.3	0.276	2	12.06
		0.2	8	6.03	21	0	2	0	0	1	6.03
						0	3	0	0	1	6.03
					22	2	1	0.1	0.123	2	12.06
					23	4	1	0.2	0.202	2	12.06
					24	6	1	0.3	0.281	2	12.06
	200	0.1	8	6.03	25	0	3	0	0	2	12.06
						0	2	0	0.018	1	6.03
					26	2	1	0.1	0.125	2	12.06
					27	4	1	0.2	0.211	2	12.06
					28	6	1	0.3	0.274	2	12.06
									0.284	1	6.03
		0.2	14	5.28	29	0	3	0	0	2	10.56
			12	4.52	30	2	1	0.1	0.125	1	4.52
			14	5.28						1	5.28
			12	4.52	31	4	1	0.2	0.242	2	9.05
			12	4.52	32	6	1	0.3	0.272	2	9.05
**Total**										34	195.28

Nine cutting inserts were used for the experiments. Table 5 summarises the usage details by cutting conditions, including the number of experiments per insert and edge and the minimum, mean, and maximum *VB* values recorded. The same tool and edge were reused for various conditions and, in some cases, materials. Reusing the same tool across a range of conditions and materials can offer several advantages. First, it isolates the impact of cutting conditions and material properties on wear progression, eliminating tool variation as a factor. Second, utilising the same tool for various conditions can provide insight into how wear patterns and rates change with different parameters, and thus offer a deeper understanding of tool-material interactions and wear mechanisms. This could be beneficial for transfer and continual learning approaches in DL-based TCM for industrial applications, as it allows DL models to leverage knowledge gained from one set of conditions to more effectively adapt to new situations.

**Table 5:** Experiment count by cutting conditions.

Cutting insert	Edge	Material	Min *VB*	Mean *VB*	Max *VB*	Experiments	Count
0	2	Cast iron GG30	0	0.006	0.018	17, 21, 25	3
	3	Cast iron GG30	0	0	0	17, 21, 25, 29	6
1	1	Stainless steel 316L	0.132	0.136	0.139	2, 6, 10, 14	8
2	1	Cast iron GG30	0.12	0.123	0.125	18, 22, 26, 30	8
3	1	Stainless steel 316L	0.202	0.206	0.213	3, 7, 11, 15	9
4	1	Cast iron GG30	0.201	0.214	0.242	19, 23, 27, 31	8
6	1	Cast iron GG30	0.272	0.277	0.284	20, 24, 28, 32	9
		Stainless steel 316L	0.287	0.289	0.291	4, 8, 12, 16	8
9	1	Stainless steel 316L	0	0.015	0.03	1, 5	4
	2	Stainless steel 316L	0	0.0195	0.039	9, 13	4

### Data acquisition

**Fig. 7:**
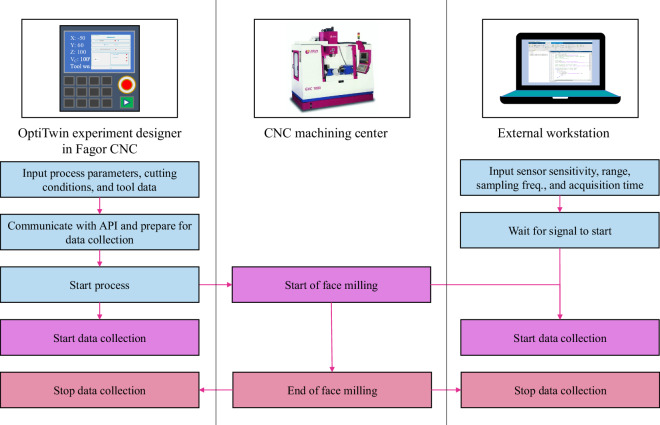
Data acquisition flow.

The data acquisition process and the interaction between components are illustrated in Fig. 7. To capture external signals, a MATLAB script was executed in the external workstation that interfaced with the DAQ unit. This script configured the acquisition parameters (sensor sensitivity, range, sampling frequency, and acquisition time) of the DAQ unit. The script then waited for a trigger from the CNC, which was an analogous output configured to indicate when the process had started. Once the triggering signals were received, the data acquisition started until the acquisition time elapsed and the face milling stopped.

Running directly on the CNC, the OptiTwin experiment designer acquired the internal CNC signals using a custom API within the CNC software. This ensured seamless integration with the CNC system and its inherent sampling rate of 250 Hz. In addition, experiment definition data, i.e., process data, cutting conditions, and tool data, were entered in the OptiTwin experiment designer. Specifically, the following data were recorded:

- **(a) Process data**: Experiment identifier, repetition number, process type (milling, drilling, and turning), machine, workpiece material, lubrication type.
- **(b) Cutting conditions**: Cutting speed (*v*_*C*_), feed rate (*f*), axial depth of cut (*a*_*p*_), and radial depth of cut (*a*_*e*_).
- **(c) Tool data**: Tool identifier, diameter, material, coating, manufacturer, part reference number, and initial *VB*.

The sampling rates for signal acquisition were chosen based on the capacity of the setup, with the aim of acquiring the highest quantity of data. Internal CNC signals were sampled at 250 Hz, which was limited by the sampling capabilities of the CNC system. However, for external sensors that measure vibration, force, and AE signals, higher sampling rates were employed to capture more detailed information about tool-material interaction. Vibration and force signals were acquired at 50 kHz, while AE signals were sampled at a higher rate of 1 MHz, capturing high-frequency information about cutting dynamics, tool wear progression, and potential anomalies. The sampling rates were maintained consistent across all experiments to ensure data comparability and facilitate further analysis.

Synchronisation issues arose between the CNC machine and the external workstation, due to technical limitations in communication between the CNC machine and the external workstation, resulting in unsynchronised internal and external signals. This is further detailed in the “Data Synchronisation” subsection. Due to this issue, four experiments did not capture external signals during the first repetition, as the MATLAB script was not manually initiated. Specifically, for cutting insert 3 with edge 1, at cutting conditions of *v*_*C*_ 50 m/min and *f* 0.05 mm/rev with an approximate *VB* of 0.2 mm, and for insert 6 with edge 1, at *v*_*C*_ 200 m/min and *f* 0.1 mm/rev with an approximate *VB* of 0.3 mm, a third repetition was conducted to ensure that at least two complete repetitions with external signals were available. In the case of insert 9 with edge 2, for *v*_*C*_ 100 m/min and feed rates *f* 0.05 and 0.1 mm/rev, where the *VB* was close to 0.0 mm, an additional repetition was not performed to avoid altering the *VB* condition.

#### Data annotation

**Fig. 8:**
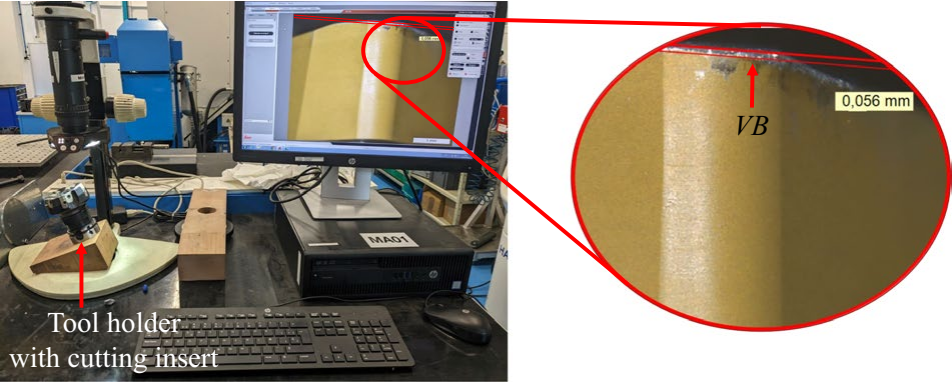
Example of *VB* measurement.

Tool *VB* was measured with the macroscope at the beginning of each experiment, to 3 decimal places (Fig. 8). Images were captured at 2x zoom level. Two images were captured per measurement, one with annotation and the other without. The unannotated images offer researchers the opportunity to implement varying image processing and annotation techniques, free from pre-existing markings. The images were then stored alongside the acquired sensor data in the experiment manager. The value was also recorded in the OptiTwin experiment designer subsystem in the CNC, linking it to the corresponding experiment.

#### Data storage

The internal signals, acquired by the OptiTwin experiment designer on the CNC machine, were transmitted via TCP sockets to the centralised OptiTwin experiment manager. There, the data were stored in a custom comma-separated-value (CSV) file format, which includes a header that records the *VB*, process data, and cutting conditions, all collected by the experiment designer.

The external signals were stored during acquisition directly on the external workstation using the MATLAB script. Upon completion, the script stored the acquired signals and their corresponding timestamps in MATLAB-formatted data files (.mat extension), facilitating efficient data access and analysis. The files were subsequently uploaded to the OptiTwin experiment manager to ensure centralised storage and seamless integration of both internal and external data within the OptiTwin framework for comprehensive analysis.

### Data synchronisation

Internal sensor data were synchronised with the external sensor data. This synchronisation ensured comprehensive monitoring of the machining process by correlating internal machine states with external measurements.

Automatic synchronisation was not possible due to technical limitations in communication between the CNC machine and the external workstation. Additionally, delays were observed between the cutting force and vibration AE signals, probably caused by the preprocessing through the piezotron coupler. As a result, the data were synchronised manually to ensure accurate alignment between the signals, as described in the “Technical Validation” section.

First, the internal signals and the experiment definition data were merged into the external signals files, maintaining MATLAB formatting for consistency. Then, synchronisation was carried out by analysing both sets of signals to identify initial and final peaks, which corresponded to the entry and exit of the tool in the workpiece. To ensure alignment, the first peak was skipped to synchronise when the tool had fully entered the workpiece, as the initial peak is small until full entry. Since the signals reflect different aspects of the process, such as spindle speed, cutting forces, and vibration, slight variations in peak location were expected as a result of tool-material interaction dynamics.

The internal SREAL signal (actual spindle speed) was used as the reference, as it showed distinct markers when the spindle speed changed at the entry and exit points of the tool. For external signals, the cutting force on the Z axis (Fz) was chosen as reference for synchronisation, despite the fact that the cuts were performed horizontally along the *X* axis. This was because material resistance causes the tool to exert a downward force on the Z axis as it engages the material (enters the cut). Similarly, as the tool exits the material, there is a release of resistance, resulting in a decrease in the force along the Z axis. These distinct changes in the *Z* axis force provided clear and sharp peaks, making it ideal for synchronisation. Moreover, the AE_RMS signal was aligned with Fz, as both signals accurately captured the entry and exit points. This approach effectively synchronised the internal and external datasets, ensuring accurate analysis of the machining process.

**Fig. 9:**
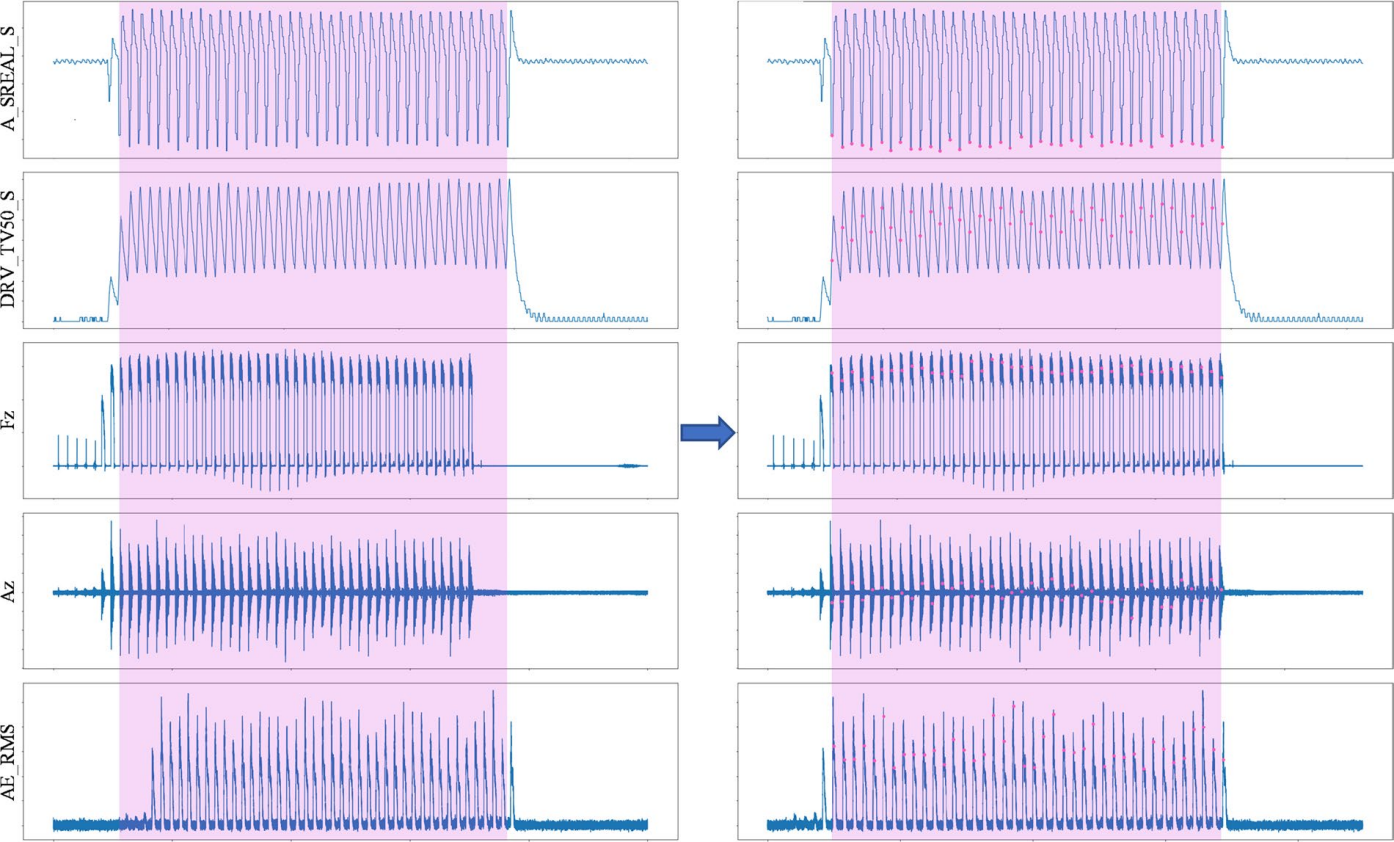
Example of the signal synchronisation process.

Figure 9 illustrates an example of the signal synchronisation process. On the left of the figure, the unsynchronised signals are shown. From top to bottom, the first two signals correspond to the internal signals: SREAL and TV50 (spindle motor power feedback), while the following three are the external signals: Fz (cutting force on the *Z* axis), Az (vibration in the *Z* axis), and AE_RMS (RMS acoustic emission). In this unsynchronised view, it is evident that the external and internal signals are misaligned, and the AE_RMS signal displays a noticeable delay compared to the other external signals. On the right of the figure, the signals are presented after synchronisation. It can be observed that both internal and external signals are now properly aligned, ensuring accurate correlation between the measurements.

## Data Records

The MU-TCM face-milling dataset is available at the digital repository (eBiltegia) of MU^15^, with this section being the primary source of information on the availability and content of the data being described. Due to the large size of the dataset, a smaller subset has been included in the digital repository to allow users to evaluate the data before committing to downloading the full dataset. This subset includes the data for experiments 13 to 20, covering both materials under identical cutting conditions. The dataset and the subset are both organised into three main folders: (i) unsynchronised signals, (ii) synchronised signals, and (iii) *VB* images.

**Fig. 10:**
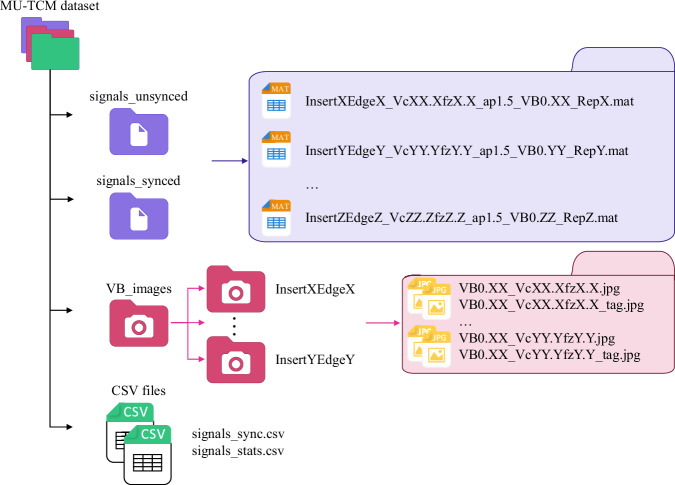
Folder structure of the MU-TCM dataset.

The unsynchronised and synchronised signals folders are composed of files with .mat extension, which include both external and internal signals, as well as the experiment definition data. The *VB* images folder is organised into subfolders, with each folder corresponding to a specific cutting insert and edge. These subfolders contain images of the measured *VB* for each experiment. In addition to the three folders, two CSV files with .csv extension are provided: (i) signal synchronisation details and (ii) cutting conditions and extracted features in time, frequency, and time-frequency domains. Figure 10 illustrates the folder structure of the MU-TCM dataset.

In the unsynchronised and synchronised signals folders, the files are named using a combination of the cutting insert number, edge number, cutting conditions, and repetition number for each experiment. For example, for the first repetition of the cutting insert 2 and edge 1, with a *v*_*C*_ of 200 m/min, a *f* of 0.2 mm/rev, a *a*_*p*_ of 1.5 mm and a *VB* of 0.125 mm, the file name would be:

**Insert2Edge1_Vc200.0_fz0.2_ap1.5_VB0.125_Rep1.mat**.

The *VB* images are stored in subfolders, which are named based on the cutting insert and edge numbers. Each subfolder contains two images with “.jpg” extension per experiment: one with annotated *VB* values and one without, together with images of a final measurement for each cutting edge and insert. The images are named using a combination of *VB* and cutting conditions, as well as a tag label for the annotated images. For example, for the measurement of the cutting insert 3 and edge 1, with a *v*_*C*_ of 50 m/min, a *f* of 0.05 mm/rev, and a *VB* of 0.202 mm, the file names would be:

**Insert3Edge1\VB0.202_Vc50.0_fz0.05.jpg**.

**Insert3Edge1\VB0.202_Vc50.0_fz0.05_tag.jpg**.

The images for the final measurement are named using a combination of *VB* and an end tag, as well as a tag label for the annotated images. For example, for the final measurement of the cutting insert 3 and edge 1, with a *VB* of 0.213 mm, the file names would be:

**Insert3Edge1\VB0.213_end.jpg**.

**Insert3Edge1\VB0.202_end_tag.jpg**.

Finally, the two CSV files are located at the root of the dataset structure. The file for signal synchronisation is named signals_sync.csv and the file for cutting conditions and extracted features is named signals_stats.csv.

### Data organisation of unsynchronised and synchronised signals

The data are stored in files with .mat extension, which organises information in a dictionary-like structure. These files contain both internal and external signals, as well as the experiment definition data. Table 6 details the keys and their corresponding values (datatype, logical type, description, and unit).

**Table 6:** Organisation of the unsynchronised and synchronised signals files.

Key	Value datatype	Logical type	Value description	Unit
ae	float64	Experiment definition	Radial depth of cut (*a*_*e*_). Always has the value 58.5.	mm
AE_F	Array of float64	External signal	Filtered accoustic emision signal.	V
AE_F	Array of float64	External signal	RMS accoustic emision signal.	V
ap	float64	Experiment definition	Axial depth of cut (*a*_*p*_). Always has the value 1.5.	mm
A(x-y-z)	Array of float64	External signal	Vibrations (gravitational acceleration) in three axes.	g
CV3_(S-X-Y-Z)	Array of float64	Internal signal	RMS current feedback of the spindle motor and table motors in three axes.	Arms
Date	string	Experiment definition	Date and time of the start of the experiment with format (yyyy-MM-dd hh:mm:ss).	N/A
fz	float64	Experiment definition	Feed rate (*f*) per tooth.	mm/rev
FREAL	Array of float64	Internal signal	Actual *f*.	mm/rev
F(x-y-z)	Array of float64	External signal	Cutting forces in three axes.	N
ID	string	Experiment definition	Experiment identifier.	N/A
Insert	int	Experiment definition	Cutting insert numbers.	N/A
Edge	int	Experiment definition	Edge number of Insert.	N/A
Lubrication	string	Experiment definition	Type of lubrication. Possible values are Dry or MQL.	N/A
Machine	string	Experiment definition	Machine make and model. Always has the value Lagun GVC 1000-HS.	N/A
POS_S	Array of float64	Internal signal	Spindle angular position within a 360^∘^ rotation.	^∘^
POS_(X-Y-Z)	Array of float64	Internal signal	Spindle position relative to the machine table in three axes.	*μ*m
Repetition	string	Experiment definition	Number of repetition.	N/A
SREAL	Array of float64	Internal signal	Actual spindle speed.	RPM
ToolDiameter	float64	Experiment definition	Diameter of the tool holder. Always has the value 80.0.	mm
ToolID	string	Experiment definition	Tool holder identifier. Always has the value Plato D80 Z1.	N/A
ToolManufacturer	string	Experiment definition	Manufacturer of the cutting insert. Always has the value AYMA.	N/A
ToolMaterial	string	Experiment definition	Material of the cutting insert. Always has the value HM.	N/A
ToolReference	string	Experiment definition	Reference of the cutting insert. Always has the value SPKR 1203EDSRM55 AF720.	N/A
TV2_(S-X-Y-Z)	Array of float64	Internal signal	Motor torque feedback of the spindle motor and table motors in three axes.	Nm
TV50	Array of float64	Internal signal	Spindle motor power feedback.	kW
TV51	Array of float64	Internal signal	Spindle motor active power.	W
VB	float64	Experiment definition	Tool wear (*VB*) measured before the experiment beginning.	mm
Vc	float64	Experiment definition	Cutting speed (*v*_*C*_).	m/min
WorkpieceMaterial	string	Experiment definition	Material of the workpiece.	N/A

### Data organisation of *VB* images

The *VB* images data are organised into subfolders corresponding to each cutting insert and edge. In each folder, images are stored for every experiment execution, with two images per experiment: one showing the annotated *VB* measurement and one without annotations. Additionally, each folder contains a final set of images taken for each cutting edge and insert at the conclusion of the experiment. All images are stored in JPEG format (.jpg extension) for ease of access and analysis.

### Data organisation of CSV files

The files are structured in a CSV format, with each row corresponding to a specific experiment file. Values are separated by semicolons (;). The signals_sync.csv file contains key reference points and values identified during the manual signal synchronisation process. The signals_stats.csv file summarises cutting conditions, as well as time, frequency, and time-frequency domain features extracted for each signal of the experiments. The extracted features are based on the methodology proposed by Wang *et al*.^16^ for training ML models for TCM. Detailed steps for signal synchronisation and feature extraction are outlined in the “Technical Validation” section. The files are organised as follows:

- signal_sync.csv:
  - file_name: Name of the signals file.
  - RPM_avg: Average value calculated from the SREAL signal.
  For the internal (i) and external signals (e1 for signals at 50 kHz and e2 for signals at 1 MHz),
  - (e1-e2-i)_signal: Signals selected as reference for synchronisation.
  - (e1-e2-i)_start: The start index of the signals selected after synchronisation.
  - (e1-e2-i)_end: The end index of the signals selected after synchronisation.
  - (e1-e2-i)_peak_distance: The average distance between peaks.
  - (e1-e2-i)_freq_peaks: The calculated frequency of the signals. This is calculated as *RPM*_*avg* ÷ 60 × *peak*_*dist*, where peak_dist is the value of peak_distance.
  - (e1-e2-i)_peak_first: The index of the first peak.
  - (e1-e2-i)_peak_last: The index of the last peak.
  - (e1-e2-i)_peak_qty: The quantity of peaks between start and end.
  - (e1-e2-i)_peak_height: The minimum height (value) to identify peaks selected during the synchronisation process.
  - (e1-e2-i)_peaks_value_avg: The average value of the peaks.
  - (e1-e2-i)_peaks_value_max: The maximum value of the peaks.
  - (e1-e2-i)_peaks_value_min: The minimum value of the peaks.
  For the e1 and e2 signals,
  - (e1-e2)_sec_search: Number of seconds of the start of the signal selected to look for the first peak and to identify (e1-e2)_peak_height.
  - (e1-e2)_sec_between_peaks: Number of seconds of the start of the signal selected to look for the first peak and to identify (e1-e2)_peak_height.
- signals_stats.csv:
  - file_name: Name of the signals file.
  - RPM_avg: Average value calculated from the SREAL signal.
  - material: Workpiece material.
  - VB: The *VB* measured before the beginning of the experiment.
  - Vc: The *v*_*C*_ of the experiment.
  - ae: The *a*_*e*_ of the experiment.
  - ap: The *a*_*p*_ of the experiment.
  - fz: The *f* per tooth of the experiment.
  For each signal,
  - (signal)_start): Indicates the start index of the signal used to extract the features.
  - (signal)_end):Indicates the end index of the signal used to extract the features.
  Time domain features:
  - (signal)_max): Indicates the maximum value.
  - (signal)_kurt): Indicates the kurtosis value.
  - (signal)_rms): Indicates the RMS value.
  - (signal)_skew): Indicates the skewness value.
  - (signal)_var): Indicates the variance value.
  - (signal)_ptp): Indicates the peak-to-peak value.
  Frequency domain features:
  - (signal)_speckurt): Indicates the spectral kurtosis value.
  - (signal)_specskew): Indicates the spectral skewness value.
  Time-frequency domain feature:
  - (signal)_wavenergy): Indicates the wavelet energy value.

## Technical Validation

The technical validation of the MU-TCM dataset focused on signal accuracy and reliability. The first stage of validation involved checking the synchronisation of internal and external signals. As mentioned in the “Data synchronisation” subsection, technical limitations in communication between the CNC machine and the external workstation meant that the signals were not automatically synchronised. Therefore, a manual signal synchronisation process was carried out based on peak analysis. This approach ensured that key reference points in both the internal and external signals were aligned, facilitating accurate correlation across datasets. The steps applied to synchronise the signals were the following:

1. **Initial visual inspection**: Internal and external signals were loaded and plotted to visually inspect the misalignment level. The user was prompted to optionally trim the external signals, as they often contained additional noise at the end due to the lack of automatic synchronisation between the CNC and external workstation. This trimming removed any recorded noise after the experiment had ended.
2. **Selection of reference internal signal**: The internal signal to be used as the reference for synchronisation was selected. The SREAL signal, representing the actual spindle RPM, was suggested by default.
3. **Peak identification in internal signals**: The peaks in the selected internal signal were identified using the find_peaks function from the SciPy library. The minimum height parameter for peak detection was set manually by the user to ensure accurate peak selection. Given the noisy nature of the signals, the find_peaks function often detected neighbouring local peaks. To compensate for this, an average of neighbouring peaks was calculated to determine a stable reference point for synchronisation.
4. **Selection of reference 50 kHz external signal**: The external signal sampled at 50 kHz to be used as reference was then selected, with the cutting forces in the Z-axis (Fz) suggested by default. The user was asked to specify how many seconds of the start of the signal should be analysed to identify the appropriate minimum peak height.
5. **Peak identification in 50 kHz external signals**: Similar to the internal signal, the peaks in the 50 kHz external signal were identified using the find_peaks function. Again, noisy signals were managed by averaging neighbouring peaks to provide a reliable synchronisation reference.
6. **Calculation of trimming signal portions**: Since the internal signals were sampled at a lower frequency than the external signals, the time difference between the start of the acquisition and the first peak was adjusted proportionally using the ratio of their respective sampling frequencies. The same was done for the time difference between the last peak and the end of the acquisition. Based on this ratio, the cutoff sections of the external signals at the start and end were calculated to align with the internal signal, ensuring that both sets of signals matched in time.
7. **Synchronisation of 1 MHz signals**: Since the AE signals had acquisition delays due to filtering, steps 5 and 6 were repeated for these signals, using AE_RMS as reference.
8. **Final signal alignment**: After identifying the trimming sections of both the internal and external signals, the signals were synchronised in time. This was validated by plotting the signals, as shown in Fig. 9.

After synchronisation, the next step involved extracting time, frequency, and time-frequency features from the signals to evaluate trends and behaviours in the machining process. The features to be extracted were defined following the methodology proposed by Wang *et al*.^16^ to train ML models for TCM. Table 7 summarises the extracted features.

**Table 7:** Features extracted from the MU-TCM dataset.

Domain	Feature	Abbreviation in the CSV file
Time	Root-mean-squared	RMS
	Variance	var
	Maximum	max
	Kurtosis	kurt
	Skewness	skew
	Peak-to-peak	ptp
Frequency	Spectral skewness	specskew
	Spectral kurtosis	speckurt
Time-frequency	Wavelet energy	wavenergy

For each experiment, the extracted features were analysed against the corresponding *VB* measurements to identify correlated or inversely correlated trends. Pearson and Spearman correlation coefficients were calculated to quantify the strength and direction of these relationships. Features showing significant correlations with *VB* values were identified, providing insight into the potential of these signals for monitoring tool wear during the machining process.

Table 8 presents the correlation coefficients between the extracted features of external signals and the *VB* for cast iron and stainless steel, and Table 9 presents the same for the extracted features of internal signals. In the case of the external signals, correlations between the extracted features and the *VB* values varied across the signals, with both positive and negative relationships observed. Correlations were consistently strong across several time-domain and frequency-domain features. In particular, for the cutting force signals, strong correlations (above 0.8) were found for RMS, variance, peak-to-peak (ptp), and wavelet energy features in Fx and Fz, with slightly lower but still strong correlations in Fy (0.75 to 0.89). This is in agreement with the existing literature, in which cutting force sensors have been effectively used to monitor machining processes^4^. In contrast, correlations for the AE signals were generally lower and more variable. AE_F displayed a moderate to strong negative correlation with VB for SS in spectral skewness and wavelet energy (around -0.7), while a positive correlation (0.77) was observed for CI in the frequency-domain features for SS. In the vibration signals, weaker correlations were identified.

**Table 8:** Correlation coefficients between extracted features of external signals and *VB* for cast iron (CI) and stainless steel (SS).

Signal	Material	Coefficient	RMS	var	max	kurt	skew	ptp	spec kurt	spec skew	wav energy
AE_F	CI	Pearson	0.00	0.00	0.07	0.32	− 0.55	0.04	0.77	0.77	− 0.21
		Spearman	0.07	0.07	0.17	0.08	− 0.59	0.18	0.77	0.77	− 0.24
	SS	Pearson	0.02	0.05	0.07	− 0.27	0.15	0.04	− 0.76	− 0.74	− 0.63
		Spearman	0.04	0.32	0.11	− 0.13	0.25	0.10	− 0.69	− 0.65	− 0.44
AE_RMS	CI	Pearson	0.01	− 0.07	0.03	0.41	0.33	− 0.13	0.29	0.28	0.00
		Spearman	0.08	0.06	0.15	0.48	0.14	− 0.03	0.41	0.42	0.08
	SS	Pearson	0.09	0.03	− 0.04	0.31	0.32	− 0.05	0.12	0.12	0.05
		Spearman	0.30	0.29	0.15	0.19	0.17	0.13	0.32	0.33	0.30
Ax	CI	Pearson	0.33	0.26	0.17	− 0.47	0.16	0.13	0.21	0.26	0.38
		Spearman	0.41	0.41	0.30	− 0.41	0.17	0.27	0.15	0.16	0.51
	SS	Pearson	0.25	0.20	− 0.02	− 0.27	0.29	− 0.25	− 0.54	− 0.53	0.51
		Spearman	0.39	0.39	− 0.05	− 0.26	− 0.01	− 0.13	− 0.56	− 0.54	0.64
Ay	CI	Pearson	0.52	0.52	0.40	− 0.34	0.17	0.40	0.31	0.35	0.69
		Spearman	0.53	0.53	0.38	− 0.38	0.10	0.39	0.23	0.23	0.71
	SS	Pearson	0.62	0.57	0.67	− 0.21	0.62	0.35	− 0.47	− 0.44	0.53
		Spearman	0.73	0.73	0.83	0.45	0.72	0.65	− 0.35	− 0.26	0.64
Az	CI	Pearson	− 0.02	− 0.06	− 0.07	− 0.03	0.32	− 0.06	0.02	− 0.12	− 0.20
		Spearman	0.10	0.10	0.02	0.04	0.40	0.05	− 0.05	− 0.12	− 0.01
	SS	Pearson	0.25	0.18	0.15	− 0.26	0.29	− 0.02	− 0.44	− 0.36	0.58
		Spearman	0.36	0.36	0.42	0.24	0.23	0.27	− 0.38	− 0.35	0.81
Fx	CI	Pearson	0.90	0.88	0.65	− 0.71	− 0.07	0.83	0.10	0.10	0.80
		Spearman	0.89	0.89	0.65	− 0.71	− 0.04	0.82	0.29	0.29	0.83
	SS	Pearson	0.90	0.86	0.85	− 0.47	0.45	0.87	0.07	0.07	0.88
		Spearman	0.95	0.95	0.86	− 0.61	0.56	0.92	0.12	0.12	0.95
Fy	CI	Pearson	0.75	0.76	0.61	− 0.40	0.34	0.74	0.06	0.06	0.73
		Spearman	0.74	0.80	0.75	− 0.48	0.45	0.74	0.14	0.14	0.72
	SS	Pearson	0.81	0.79	0.26	− 0.25	0.19	0.78	− 0.32	− 0.33	0.78
		Spearman	0.89	0.90	0.39	− 0.18	0.11	0.83	− 0.56	− 0.56	0.89
Fz	CI	Pearson	0.94	0.89	0.93	− 0.61	− 0.46	0.95	− 0.17	− 0.17	0.89
		Spearman	0.95	0.96	0.94	− 0.66	− 0.48	0.95	− 0.21	− 0.21	0.95
	SS	Pearson	0.93	0.88	0.94	− 0.35	− 0.35	0.92	− 0.61	− 0.62	0.89
		Spearman	0.98	0.99	0.99	− 0.51	− 0.47	0.97	− 0.57	− 0.59	0.98

**Table 9:** Correlation coefficients between extracted features of internal signals and *VB* for cast iron (CI) and stainless steel (SS).

Signal	Material	Coefficient	RMS	var	max	kurt	skew	ptp	spec kurt	spec skew	wav energy
CV3_X	CI	Pearson	0.79	0.77	0.83	0.29	0.56	0.83	0.39	0.40	0.09
		Spearman	0.83	0.83	0.86	0.35	0.47	0.86	0.32	0.36	− 0.01
	SS	Pearson	0.77	0.72	0.66	0.17	0.57	0.66	0.36	0.35	0.11
		Spearman	0.86	0.87	0.77	0.34	0.63	0.77	0.25	0.29	0.08
CV3_Y	CI	Pearson	− 0.57	0.41	0.08	− 0.53	0.60	0.34	− 0.41	− 0.40	− 0.57
		Spearman	− 0.59	0.34	− 0.11	− 0.44	0.59	0.19	− 0.38	− 0.39	− 0.67
	SS	Pearson	0.74	0.69	0.75	− 0.20	− 0.23	0.75	− 0.20	− 0.19	0.61
		Spearman	0.76	0.80	0.80	− 0.11	− 0.31	0.80	− 0.24	− 0.27	0.71
CV3_Z	CI	Pearson	0.15	− 0.09	− 0.48	− 0.28	− 0.50	− 0.47	− 0.44	− 0.52	0.00
		Spearman	0.04	− 0.09	− 0.40	− 0.11	− 0.42	− 0.35	− 0.57	− 0.57	0.05
	SS	Pearson	0.35	0.54	0.38	− 0.31	− 0.24	0.40	− 0.14	− 0.19	0.22
		Spearman	− 0.02	0.34	0.28	− 0.17	− 0.16	0.30	− 0.21	− 0.20	− 0.02
TV2_S	CI	Pearson	0.50	0.45	− 0.05	− 0.30	0.19	0.44	0.01	0.09	0.59
		Spearman	0.42	0.42	− 0.07	− 0.38	0.25	0.38	− 0.07	0.01	0.55
	SS	Pearson	0.60	0.58	0.29	− 0.34	0.19	0.59	− 0.16	− 0.10	0.29
		Spearman	0.61	0.61	0.36	− 0.46	0.35	0.56	− 0.20	− 0.13	0.45
TV2_X	CI	Pearson	0.79	0.88	0.36	0.44	− 0.84	0.83	− 0.25	− 0.26	− 0.05
		Spearman	0.83	0.89	0.35	0.48	− 0.87	0.83	− 0.30	− 0.30	− 0.09
	SS	Pearson	0.76	0.73	0.40	− 0.31	− 0.44	0.71	− 0.16	− 0.17	0.15
		Spearman	0.83	0.82	0.10	0.17	− 0.64	0.78	− 0.19	− 0.22	0.01
TV2_Y	CI	Pearson	− 0.57	0.59	0.07	− 0.33	0.29	0.63	− 0.30	− 0.29	− 0.55
		Spearman	− 0.61	0.56	− 0.13	− 0.26	0.29	0.57	− 0.26	− 0.18	− 0.63
	SS	Pearson	0.74	0.67	0.75	0.47	− 0.37	0.78	− 0.21	− 0.24	0.63
		Spearman	0.76	0.76	0.80	0.53	− 0.54	0.85	− 0.31	− 0.31	0.75
TV2_Z	CI	Pearson	0.14	0.11	− 0.45	− 0.02	− 0.13	0.21	− 0.21	− 0.31	− 0.13
		Spearman	0.01	0.01	− 0.28	− 0.06	− 0.08	0.41	− 0.36	− 0.37	− 0.03
	SS	Pearson	0.36	0.31	0.35	− 0.28	− 0.13	0.62	− 0.19	− 0.18	0.23
		Spearman	− 0.01	− 0.03	0.10	− 0.01	− 0.06	0.87	− 0.24	− 0.21	0.01
TV50	CI	Pearson	0.35	0.55	0.44	0.01	− 0.01	0.45	− 0.10	− 0.07	0.40
		Spearman	0.32	0.51	0.43	0.01	− 0.01	0.43	− 0.11	− 0.08	0.35
	SS	Pearson	0.46	0.49	0.57	0.15	− 0.03	0.58	− 0.22	− 0.18	0.59
		Spearman	0.60	0.64	0.70	0.10	− 0.29	0.71	− 0.32	− 0.31	0.75
TV51	CI	Pearson	0.33	0.38	0.36	− 0.24	− 0.17	0.39	0.10	0.18	0.10
		Spearman	0.29	0.39	0.37	− 0.34	− 0.24	0.39	0.00	0.16	0.12
	SS	Pearson	0.36	0.41	0.40	− 0.22	0.28	0.42	− 0.04	0.01	0.02
		Spearman	0.50	0.53	0.53	− 0.32	0.19	0.51	− 0.09	0.00	0.13

The correlations of the internal signals were generally weaker than those of the external sensors, likely due to the loss of data quality resulting from lower sampling frequencies. Nevertheless, strong correlations were still identified. CV3_X demonstrated strong positive correlations across RMS, var, and p2p, where coefficients exceed 0.8, indicating a stable relationship. TV2_X exhibited a similarly strong correlation. CV3_Y and TV2_Y showed moderate correlations in SS, particularly in peak-to-peak and maximum values, although these signals presented weaker and sometimes moderate negative correlations in CI. The other signals exhibited weak to moderate correlations.

## Data Availability

All scripts used for synchronisation, feature extraction, and signal analysis are available at a public repository in GitHub^17^. The code is written in Python (version 3.11) and the library dependencies are listed in a requirements.txt file. The repository includes three main scripts designed for use with the MU-TCM face-milling dataset. The Signal_sync.py script synchronises internal and external signals. The Signal_feature_extraction.py script extracts features from the synchronised signals. Finally, the Signal_evaluator.py script assesses the extracted features against tool wear data. Researchers are encouraged to adapt the workflows of the scripts as needed for their specific use cases.
